# Lactational Western‐Style Fat Exposure in Mice Programs Metabolic Dysfunction‐Associated Steatotic Liver Disease (MASLD) in Male Offspring

**DOI:** 10.1002/mnfr.70468

**Published:** 2026-04-17

**Authors:** Amy Y. Zhao, Haijing Sun, Leyi Yan, Oyewale Shiyanbola, Dave Bridges, Brigid Gregg

**Affiliations:** ^1^ University of Michigan Medical School Ann Arbor Michigan USA; ^2^ Division of Endocrinology Department of Pediatrics Michigan Medicine Ann Arbor Michigan USA; ^3^ Epidemiology School of Public Health University of Michigan Ann Arbor Michigan USA; ^4^ Department of Pathology Michigan Medicine Ann Arbor Michigan USA; ^5^ Nutritional Sciences School of Public Health University of Michigan Ann Arbor Michigan USA

**Keywords:** developmental programming, lactation, MASLD, milk, Western diet

## Abstract

Metabolic dysfunction‐associated steatotic liver disease (MASLD) is the leading cause of liver disease. Early‐life exposure to maternal overnutrition during critical developmental windows, such as lactation, can predispose offspring to develop metabolic disease. We previously demonstrated that consumption of a high fat diet (60% fat) during lactation leads to the development of insulin resistance and MASLD in male offspring. However, no studies have investigated the role of a lactational Western‐style fat (45% fat) exposure, which is more clinically relevant and representative of human food consumption habits. Dams were fed either a standard chow or Western‐style fat diet from delivery through the 21‐day lactation period. At weaning, all offspring were fed standard chow until 3 months of age, when a subset from each group was challenged with an additional high‐fat stressor. Exposure to a Western‐style fat diet during lactation increased male offspring susceptibility to weight gain, obesity, and insulin resistance when fed a high fat diet in adulthood. Furthermore, these offspring demonstrated hepatomegaly and hepatic triglyceride accumulation, which may be mediated by altered lipid metabolism. These findings indicate that an indirect neonatal exposure to a Western‐style fat diet can program male offspring to develop MASLD later in life.

## Graphical abstract

Among the offspring fed with a high fat diet from 12 to 24 weeks of age, those that were exposed to a Western‐style fat diet during lactation had higher body weights, fat mass, insulin resistance, liver weights, and liver fat accumulation compared to a control diet, indicating increased susceptibility to MASLD.

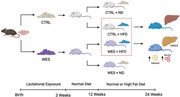

**Abbreviations**
AUC: area under the curve
CTRL: control
GTT: glucose tolerance test
GWAT: gonadal white adipose tissue
HFD: high‐fat diet
IPGTT: intraperitoneal glucose tolerance test
ITT: insulin tolerance test
IWAT: inguinal white adipose tissue
MASLD: metabolic dysfunction‐associated steatotic liver disease
ND: standard diet
PN: post‐natal
WES: Western‐style fat diet

## 1 Introduction

Metabolic dysfunction‐associated steatotic liver disease (MASLD) entails the accumulation of liver fat in association with known metabolic disease [1]. MASLD includes a spectrum of liver injury, ranging from simple steatosis to metabolic dysfunction‐associated steatohepatitis (MASH), which may lead to cirrhosis and hepatocellular carcinoma [1]. MASLD is the most common cause of chronic liver disease worldwide, with the most recent meta‐analysis estimating a global prevalence of 38% [2]. The prevalence of MASLD has been rising in parallel to that of obesity and type 2 diabetes, which are major risk factors associated with its progression [3]. In the United States, MASH is the fastest‐growing indication for liver transplantation [4]. There are currently no effective dietary interventions or medical treatments for this condition. As the burden of MASLD is projected to increase, the importance of targeting strategies to slow its progression or prevent its development is evident [4].

Dietary exposures play a role in the pathogenesis and progression of MASLD. Excess calorie intake, along with diets high in saturated fats and refined carbohydrates, which are characteristic of Western diets, promote obesity and insulin resistance. When the demand for lipid storage exceeds the capacity of existing adipocytes, lipolysis of adipose tissue occurs, resulting in an increased influx of free fatty acids to the liver [5]. Insulin resistance additionally increases de novo lipogenesis in which excess carbohydrates are converted to free fatty acids. Free fatty acids in the liver are esterified with glycerol to form triglycerides, leading to hepatic triglyceride accumulation, the hallmark of MASLD [6]. Excess fatty acids generate lipotoxic products, increasing susceptibility to hepatocellular injury, steatohepatitis, and fibrosis [6].

Maternal dietary exposures during pregnancy and lactation have the capacity to influence early‐life programming of lifelong metabolic health [7]. In particular, exposure to maternal overnutrition during critical windows of susceptibility may predispose offspring to the development of MASLD in adulthood [8, 9]. The lactation period is one of these critical windows, wherein alterations in milk composition can impact ongoing organ development and later‐life metabolic disease risk [10, 11]. In mice, a maternal high‐fat diet (HFD) confined to the lactation period has been shown to predispose offspring to insulin resistance, obesity, and hepatic steatosis [12, 13]. Previously, we demonstrated that lactational HFD exposure led offspring to develop hepatic insulin resistance and MASLD in adulthood in a sexually dimorphic manner [14].

Most studies focusing on maternal dietary exposures and overnutrition during lactation use pro‐inflammatory HFDs (60% fat). However, the human “Western‐style” diet consists of a lower percentage of total fat (35% fat) with a higher percentage of saturated and *trans*‐fat that may not be adequately represented in HFD models [15, 16]. Additionally, laboratory HFDs do not fully represent Western human food consumption habits [17]. Western‐style fat diet (WES) exposure in adult mice has been associated with insulin resistance and increased risk of developing MASLD [18, 19]. In the few studies investigating maternal WES exposure and offspring MASLD risk, maternal exposure occurred before and during both gestation and lactation [20, 21]. This reveals the combined effect of the prenatal and post‐natal (PN) environment on modulating MASLD susceptibility but does not delineate the contribution of an exposure during the lactation period. To our knowledge, no published studies have compared the effects of maternal Western‐style fats confined to lactation on metabolic liver disease outcomes in offspring. The present study investigated whether exposure to a maternal WES versus a standard chow control diet during lactation impacts the risk of developing MASLD in offspring.

## 2 Methods

### 2.1 Animal Husbandry

**FIGURE 1:**
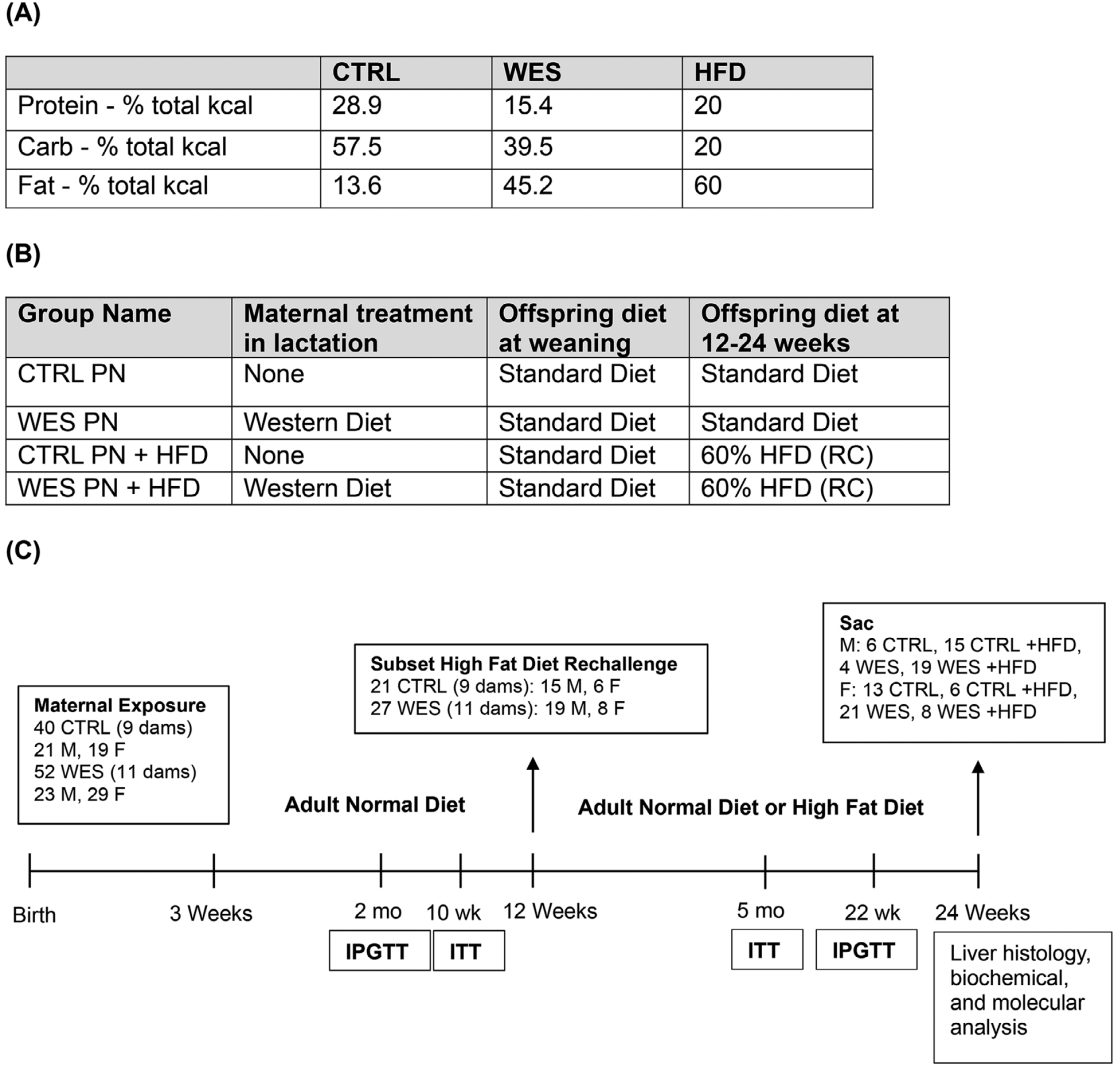
Experimental schema for experiments presented. (A) Diet composition. (B) Scheme of diet conditions per group. (C) Experimental timeline. The CTRL PN group only had normal diet. The WES PN group only had Western‐style fat diet during the lactation window. At 12 weeks of age, a subset of the offspring from each exposure group was switched to HFD while the remaining offspring continued a normal diet for 12 weeks. Numbers listed are in terms of pups and represent the highest possible number (*n*) for the timeline. In some cases, pups were omitted from analysis due to human error, bad samples, and so forth. CTRL, control; WES, Western; PN, post‐natal; HFD, high‐fat diet; IPGTT, intraperitoneal glucose tolerance test; ITT, insulin tolerance test.

Two‐month‐old Virgin C57BL/6J mice were purchased from Jackson Laboratories (Bar Harbor, ME, USA) and allowed to acclimate to the facility for two weeks before mating. Mice were kept in cages on a ventilated rack with a 12‐h light/dark cycle and provided ad libitum access to food and water. All procedures were approved by the University of Michigan Institutional Animal Care and Use Committee (Protocol Number: PRO00012189). After parturition, dams were randomly assigned to a WES diet (45.2% of calories from fat, Teklad TD.190931, Envigo, Madison, WI, USA), and control (CTRL) dams were kept on standard diet (ND) (13.5% of calories from fat, 5001 Laboratory Rodent Diet, LabDiet, St. Louis, MO, USA) for the duration of the lactation period. Our WES was modeled off the Total Western Diet (TD.110424, Envigo) but with fats proportionately increased to provide 45% of calories from fat and without the addition of fructose as would be in a traditional Western diet model. At postnatal Day 21, all pups were weaned onto the ND and housed in cages coinciding with their maternal diet groups. At 12 weeks of age, half of the pups from each litter within each diet group were randomly selected to be fed a 60% pro‐inflammatory HFD (+HFD) (D12492 Research Diets, New Brunswick, NJ, USA) as a metabolic challenge, while the rest were randomized to the ND for an additional 12 weeks. This “second‐hit” stressor is sometimes necessary to unmask programmed metabolic defects and has been employed as a metabolic challenge in our prior studies [12, 14]. Experimental offspring generated fell into four groups: maternal normal diet (CTRL PN +ND), maternal normal diet with adult HFD rechallenge (CTRL PN +HFD), maternal WES (WES PN +ND), and maternal WES with adult HFD rechallenge (WES PN +HFD). Only offspring from litters with five to nine pups were used for the experimental groups as this represented a range of average litter size in our colony (average litter size: 7). There was no difference in average litter sizes between the CTRL and WES groups. Most experiments were performed on multiple, independent cohorts to demonstrate reproducibility. Diet implementation scheme and experimental design can be seen in Figure 1.

### 2.2 Pup Tissue Collection

In one cohort using the same groups, male offspring were euthanized via CO_2_ inhalation on postnatal day 16 (P16) for blood and tissue collection. Liver, gonadal white adipose tissue (GWAT), and inguinal white adipose tissue (IWAT) were collected and weighed. Blood was collected via cardiac puncture and centrifuged for serum collection.

### 2.3 Adult Offspring Tissue Collection and Body Composition

At age 24 weeks, adult offspring were euthanized via CO_2_ inhalation. Liver, GWAT, and IWAT were collected and weighed. Blood was collected via cardiac puncture and centrifuged for serum collection. Body composition was measured by nuclear magntic resonance (NMR) at the University of Michigan Animal Phenotyping Core (MMPC‐Live) using a Minispec LF90II Brucker Optics.

### 2.4 Insulin and Intraperitoneal Glucose Tolerance Tests (ITT, IPGTT)

Insulin tolerance test (ITT) was performed on offspring at 10 and 20 weeks of age. After a 6‐h fast prior to testing, offspring received an intraperitoneal injection of insulin (0.75 units/kg for mice fed ND, 1 unit/kg for mice rechallenged with +HFD). Blood glucose values were measured from the tail vein using a glucometer at fasting (prior to injection), and at 15, 30, 60, and 90 min after injection. IPGTT was performed on offspring at 8 and 22 weeks of age. After a 6‐h fast prior to testing, offspring received an intraperitoneal injection of dextrose (2 g/kg for mice fed ND, 0.66 g/kg for mice fed +HFD). Blood glucose values were measured from the tail vein at fasting (prior to injection), and at 30, 60, and 120 min after injection. The area under the curve (AUC) was calculated for each animal as the sum of glucose values during the experiment.

### 2.5 Serum Insulin Levels

At 20 weeks of age, offspring were fasted for 6 h, and blood was collected by tail snip and centrifuged to separate the serum. Serum samples were analyzed for insulin concentration using a Mouse Ultrasensitive Insulin ELISA (80‐INSMSU‐E01, ALPCO, Salem, NH, USA).

### 2.6 Histological Liver Analysis

Liver tissue was sectioned and analyzed using Hematoxylin and Eosin (H and E) staining. Paraffin‐embedded liver sections (5 um) were deparaffinized in xylene and rehydrated in decreasing concentrations of ethanol to 95%, followed by rinsing in cold tap water. Sections were stained using a regressive Harris’ Hematoxylin Solution, rinsed with running tap water, and differentiated in 70% ethanol containing 0.1% hydrochloric acid. After two additional rinses in cold tap water, sections were placed in a Blueing solution and counterstained with 1% Eosin Y, alcoholic. Finally, sections were dehydrated with increasing concentrations of ethanol up to 100%, cleared in xylene, and mounted using Permount mounting media. Histologic features were assessed by an expert clinical pathologist using the NASH Clinical Research Network scoring system [22]. The NAFLD Activity Score (NAS) was calculated as the unweighted sum of the scores for steatosis (0–3), lobular inflammation (0–3), and ballooning (0–2).

### 2.7 Picro‐Sirius Red Staining

Picro‐sirius red staining is a histological technique used to visualize and quantify collagen fibers in tissue sections [23]. It binds specifically to collagen fibers (Collagen I and III) in formaldehyde‐fixed, paraffin‐embedded liver tissue sections and is used to assess the extent of fibrotic change by highlighting collagen accumulation within tissues. Sirius red‐stained collagen appears red under light microscopy. Paraffin‐embedded liver sections (5 um) were deparaffinized in xylene and rehydrated in 100%, 95%, 70%, and 50% ethanol. Slides were then stained in Picro‐sirius Red solution for one hour and washed twice in 0.5% acidified deionized water. Slides were dehydrated in three changes of 100% ethanol, cleared in xylene, and mounted with Permount medium. The images were taken on an Olympus microscope (Olympus Corporation of the Americas, Center Valley, PA, USA) and analyzed using freely available ImageJ software [14].

### 2.8 Hepatic Triglyceride Quantification

Total triglycerides in liver were analyzed by Thermo Scientific Triglycerides Reagent (TR22421) following the manufacturer protocol [14]. Triglycerides results were normalized to the mass of the liver tissue initially used for the assay.

### 2.9 RNA Extraction and Quantitative Real‐Time PCR (qRT‐PCR)

Ribonucleic acid (RNA) was extracted from frozen liver samples using a RNeasy Mini Kit (QIAGEN, Gaithersburg, MD, USA) according to the manufacturer's instructions. cDNA was generated using a high‐capacity cDNA reverse transcription kit (Life Technologies, Carlsbad, CA, USA), followed by analysis using real‐time PCR with Power SYBR Green PCR Master Mix (Applied Biosystems, Grand Island, NY, USA) [14]. The gene *Gapdh* was used as an internal control. A list of primer sequences can be seen in Table 1.

**TABLE 1:** Sequence for qPCR Primers.

Gene	Forward 5′‐3′	Reverse 5′‐3′
*Srebf1*	CAT CGA CTA CAT CCG CTT CTT	CAC CAG GTC CTT CAG TGA TTT
*Acaca*	ACA TTC CGA GCA AGG GAT AAG	GGG ATG GCA GTA AGG TCA AA
*Fasn*	CAA CCG GCT CTC TTT CTT CT	CCT GGT AGG CAT TCT GTA GTG
*Tnf*	TAG CCC ACG TCG TAG CAA AC	ACA AGG TAC ACC CCA TCG GC
*Fxr*	TGG CTG AAT GTA TGT ATA CAG GTT T	CAG CGT GCT GCT TCA CAT TT
*Zbtb16*	CGT CTG TGG ATC TGA ACT GTA TC	AGG AAG GAA GGA AGG AAG GA
*Gapdh*	AAC AGC AAC TCC CAC TCT TC	CCT GTT GCT GTA GCC GTA TT

### 2.10 Statistical Analysis

Unless otherwise noted, data are shown as mean ± standard error of the mean. Data were checked for normality and equal variance using the Shapiro–Wilk test. Two‐tailed *t*‐tests or Mann–Whitney *U*‐tests were used as appropriate to compare results from two groups. Two‐way ANOVA was used to analyze repeated measures of timed metabolic tests. For all tests, significance was defined as *p* < 0.05. Statistical analyses were performed using GraphPad Prism 10.1.1 software.

## 3 Results

### 3.1 Exposure to a Lactational WES Diet Alters Pup Metabolic State

**FIGURE 2:**
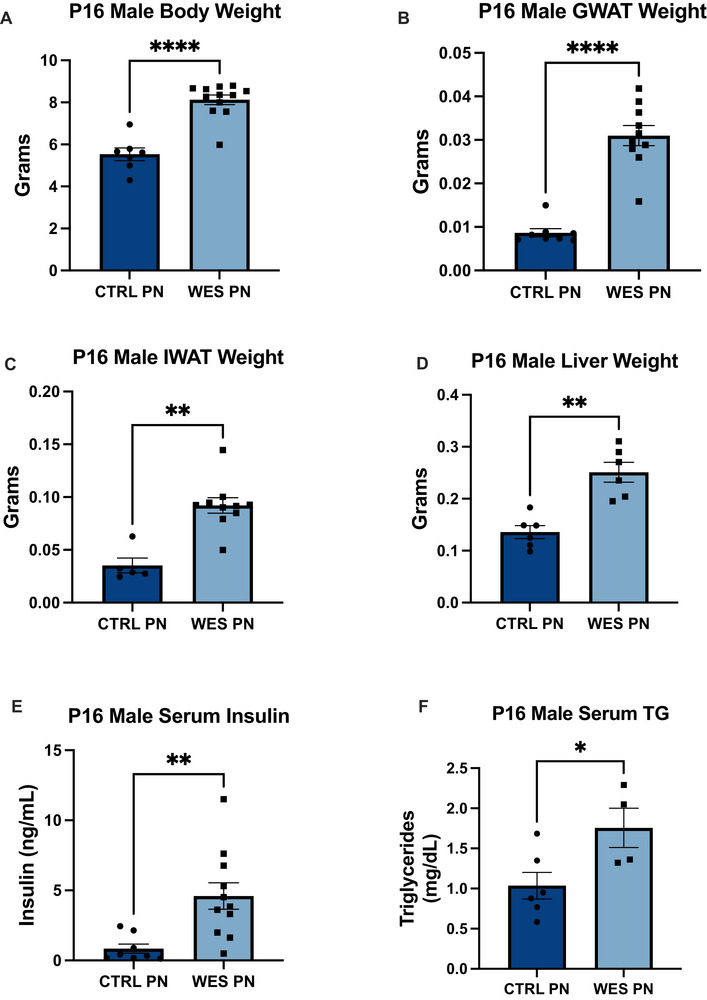
Lactational Western‐style fat diet exposure alters pup metabolic state. All black values are for CTRL PN (*n* = 5–8) and gray are for WES PN (*n* = 6–12) male offspring. (A) P16 body weight. (B) P16 gonadal white adipose tissue (GWAT) weight. (C) P16 inguinal white adipose tissue (IWAT) weight. (D) P16 liver mass. (E) P16 fed circulating insulin levels. (F) P16 circulating triglyceride levels. CTRL, control; WES, Western; PN, post‐natal. *p < 0.05, **p < 0.01, ****p < 0.0001 (unpaired *t*‐test). Data are expressed as mean ± SEM.

We first measured male pup growth and metabolic outcomes during the lactation period (P16) to determine any changes caused by maternal Western‐style fat feeding. Offspring of dams consuming a WES had higher body weights compared to offspring of CTRL dams (*p* < 0.0001, Figure 2A). These pups also had increased GWAT weight (*p* < 0.0001, Figure 2B), IWAT weight (*p* = 0.0013, Figure 2C), and liver mass (*p* = 0.0022, Figure 2D). Serum insulin (*p* = 0.0012, Figure 2E) and triglycerides (*p* = 0.0346, Figure 2F) were also increased. Overall, these results show that exposure to Western‐style fat early in life leads to an increase in pup growth.

### 3.2 Lactational Western‐Style Fat Exposure Leads to Increased Male Offspring Body Weight and Adiposity After HFD Rechallenge

**FIGURE 3:**
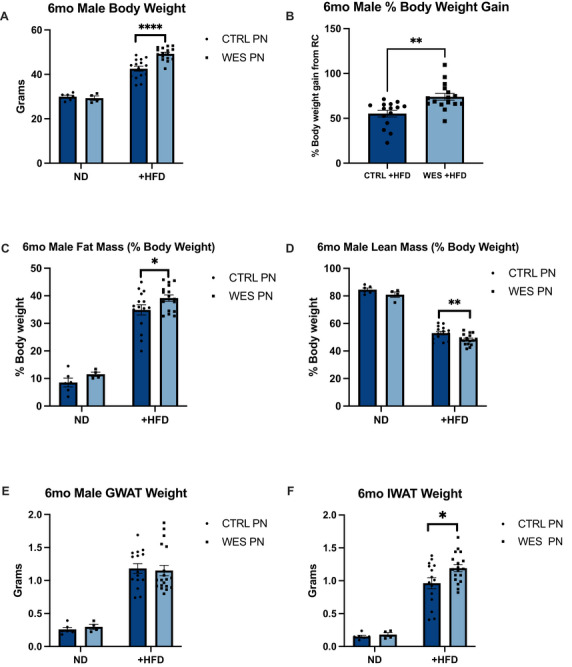
Male offspring exposed to a maternal Western‐style fat diet during lactation have altered body composition and increased inguinal white adipose tissue (IWAT) weight after HFD rechallenge at 6 months of age. (A) Body weight. (B) Percent of body weight gain from +HFD rechallenge. (C) Fat mass as percentage of body weight. (D) Lean mass as percentage of body weight. (E) Gonadal white adipose tissue (GWAT) weight. (F) Inguinal white adipose tissue (IWAT)weight. CTRL PN + ND (*n* = 6), WES PN + ND (*n* = 4), CTRL PN + HFD (*n* = 15), WES PN + HFD (*n* = 16–19). CTRL, control; WES, Western; ND, normal diet; HFD, high‐fat diet; PN, post‐natal. *p < 0.05, **p < 0.01, ***p < 0.001, ****p < 0.0001 (unpaired *t*‐test). Data are expressed as mean ± SEM.

**FIGURE 4:**
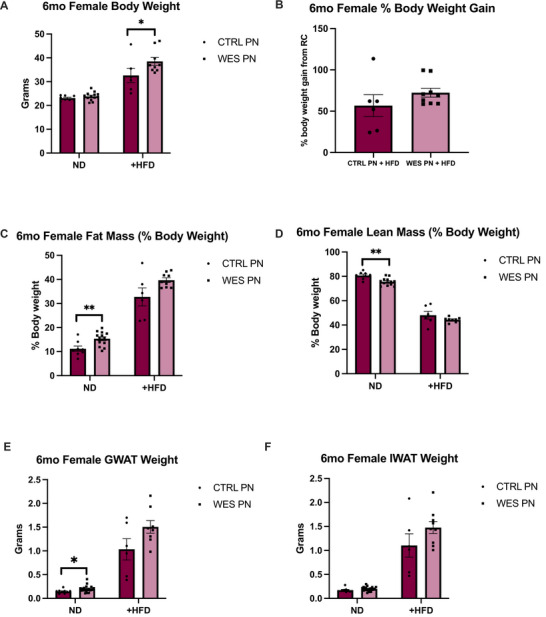
Female offspring exposed to a maternal Western‐style fat diet during lactation have increased body weight without increased adiposity after HFD rechallenge at 6 months of age. (A) Body weight: CTRL PN + ND (*n* = 8), WES PN + ND (*n* = 13), CTRL PN + HFD (*n* = 6), WES PN + HFD (*n* = 9). (B) Percent of body weight gain from +HFD rechallenge. (C) Fat mass as percentage of body weight. (D) Lean mass as percentage of body weight. (E) Gonadal white adipose tissue (GWAT) weight: CTRL PN + ND (*n* = 9), WES PN + ND (*n* = 21), CTRL PN + HFD (*n* = 6), WES PN + HFD (*n* = 9). (F) Inguinal white adipose tissue (IWAT)weight. CTRL, control; WES, Western; ND, normal diet; HFD, high‐fat diet; PN, post‐natal. *p < 0.05 (Mann–Whitney test), **p < 0.01 (unpaired *t*‐test). Data are expressed as mean ± SEM.

Individual weights were measured at Week 12 and 24 for all offspring. Within male offspring maintained on ND, there was no difference in body weight between diet groups (*p* = 0.57, Figure 3A). However, WES PN +HFD offspring had significantly higher body weights compared to CTRL PN +HFD (*p* < 0.0001, Figure 3A) and gained a higher percentage of their original body weight (*p* = 0.001, Figure 3B). Within female offspring maintained on ND, there was no difference in body weight between diet groups (*p* = 0.28, Figure 4A). WES PN +HFD offspring had significantly higher body weights compared to CTRL PN +HFD (*p* = 0.0496, Figure 4A) but did not gain more of their original body weight (*p* = 0.18, Figure 4B).

All offspring were euthanized at 6 months of age. Within male offspring maintained on ND, there was no difference in fat mass between diet groups (*p* = 0.19, Figure 3C). Body fat percentage was significantly higher among WES PN +HFD offspring compared to CTRL PN +HFD (*p* = 0.048, Figure 3C). Within male offspring maintained on ND, there was no difference in lean mass between diet groups (*p* = 0.123, Figure 3D). However, WES PN +HFD had a significantly lower lean mass as percentage of total body weight compared to CTRL PN +HFD (*p* = 0.0063, Figure 3D).

Within female offspring maintained on ND, WES PN had significantly higher fat mass compared to CTRL PN as absolute fat mass and as body fat percentage (*p* = 0.0063, Figure 4C). WES PN +HFD trended toward having higher fat mass compared to CTRL PN +HFD, but this was not statistically significant (*p* = 0.052, Figure 4C). Within female offspring maintained on ND, WES PN offspring had a lower lean mass as percentage of total body weight compared to CTRL PN offspring (*p* = 0.0012, Figure 4D). However, there was no difference in lean mass between +HFD groups (*p* = 0.215, Figure 4D).

At necropsy, GWAT and IWAT were dissected and weighed. There was no significant difference in GWAT as absolute weight or percentage of total body weight between male offspring maintained on ND and between +HFD male offspring (*p* = 0.453, Figure 3E; *p* = 0.772, Figure 3E). Within male offspring maintained on ND, there was no significant difference in IWAT between diet groups as absolute weight or percentage of total body weight (*p* = 0.414, Figure 3F). WES PN +HFD offspring had significantly higher IWAT weights compared to CTRL PN +HFD (*p* = 0.023, Figure 3F). Within female offspring maintained on ND, WES PN had significantly increased GWAT as absolute weight and percentage of total body weight compared to CTRL PN offspring (*p* = 0.011, Figure 4E). There was no significant difference in GWAT among +HFD offspring as absolute weight or percentage of total body weight (*p* = 0.081, Figure 4E). There was no difference in IWAT between ND groups or between +HFD groups when analyzed as absolute weight or percentage of total body weight (*p* = 0.088 and *p* = 0.157, respectively; Figure 4F).

### 3.3 Lactational Western‐Style Fat Exposure Results in Insulin Resistance Without Evidence of Glucose Intolerance After HFD Rechallenge in Male Offspring

**FIGURE 5:**
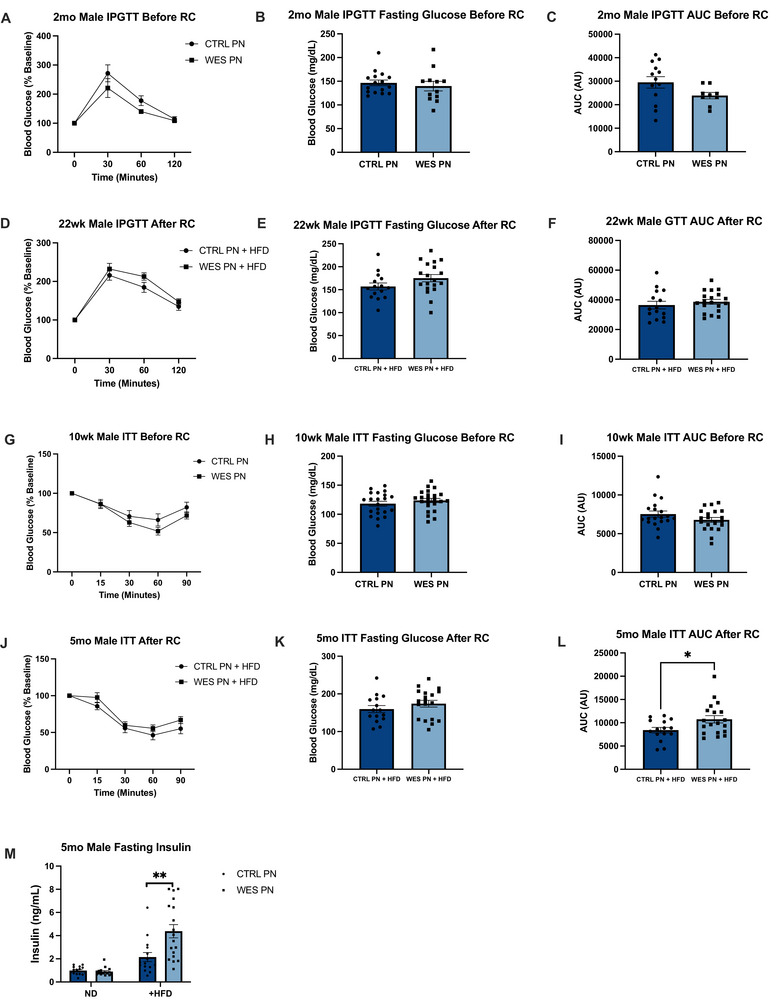
Male offspring exposed to a maternal Western‐style fat diet during lactation demonstrate insulin resistance but normal glucose tolerance after HFD rechallenge. (A) Intraperitoneal glucose tolerance test (IPGTT) performed on 2‐mo‐old offspring as percent of baseline: CTRL (*n* = 13), WES (*n* = 11). (B) Fasting blood glucose at 2 months. (C) IPGTT AUC at 2 months. (D) IPGTT performed on 22‐wk‐old offspring as percent of baseline: CTRL + HFD (*n* = 15), WES + HFD (*n* = 19). (E) Fasting blood glucose at 22 weeks. (F) IPGTT AUC at 22 weeks. (G) Insulin tolerance test (ITT) performed on 10‐wk‐old offspring as percent of baseline: CTRL (*n* = 19), WES (*n* = 20). (H) Fasting blood glucose at 10 weeks. (I) ITT AUC at 10 weeks. (J) ITT performed on 5‐mo‐old offspring as percent of baseline: CTRL + HFD (*n* = 15), WES + HFD (*n* = 19). (K) Fasting blood glucose at 5 months. (L) ITT AUC at 5 months. (M) Circulating fasting insulin levels of 2‐mo‐old male offspring before and after HFD rechallenge: CTRL PN + ND (*n* = 15), WES PN + ND (*n* = 16), CTRL PN + HFD (*n* = 15), WES PN + HFD (*n* = 19). AUC, area under the curve; CTRL, control; WES, Western; ND, normal diet; HFD, high‐fat diet; PN, post‐natal. Two‐way ANOVA was used to analyze repeated measures of timed metabolic tests. *p < 0.05, **p < 0.01 (unpaired *t*‐test). Data are expressed as mean ± SEM.

We next examined male offspring metabolic profiles before and after +HFD rechallenge given the associations between obesity, impaired glucose tolerance/insulin resistance, and MASLD. At age 2 months, average fasting blood glucose (mg/dL) was not significantly different between CTRL PN and WES PN offspring (*p* = 0.544, Figure 5B). Following dextrose injection, the average IPGTT AUC was not significantly different between CTRL PN and WES PN (*p* = 0.089, Figure 5C). At age 22 weeks, average fasting blood glucose was not significantly different between diet groups among +HFD offspring (*p* = 0.114, Figure 5E). Following dextrose injection, the average IPGTT AUC was not significantly different between diet groups (*p* = 0.455, Figure 5F).

At age 10 weeks before HFD rechallenge, average fasting blood glucose was similar between CTRL PN and WES PN offspring (*p* = 0.329, Figure 5H). Following insulin injection, there was no significant difference in average ITT AUC between diet groups (*p* = 0.146, Figure 5I). Two‐way ANOVA by diet and time did not reach statistical significance between diet groups. At 5 months of age, fasting blood glucose was similar between +HFD diet groups (*p* = 0.277, Figure 5K). Following insulin injection, the average ITT AUC was significantly higher in WES PN + HFD offspring (*p* = 0.03, Figure 5L), indicating insulin resistance.

There was no difference in fasting serum insulin between CTRL PN and WES PN offspring at 2 months of age (*p* = 0.459, Figure 5M). Insulin levels were significantly higher in WES PN +HFD compared to CTRL PN +HFD offspring at 2 months after HFD rechallenge (*p* = 0.0042, Figure 5M).

### 3.4 Lactational Western‐Style Fat Exposure Leads to Increased Liver Weight, Triglyceride Accumulation, and Evidence of Hepatic Steatosis After HFD Rechallenge in Male Offspring

**FIGURE 6:**
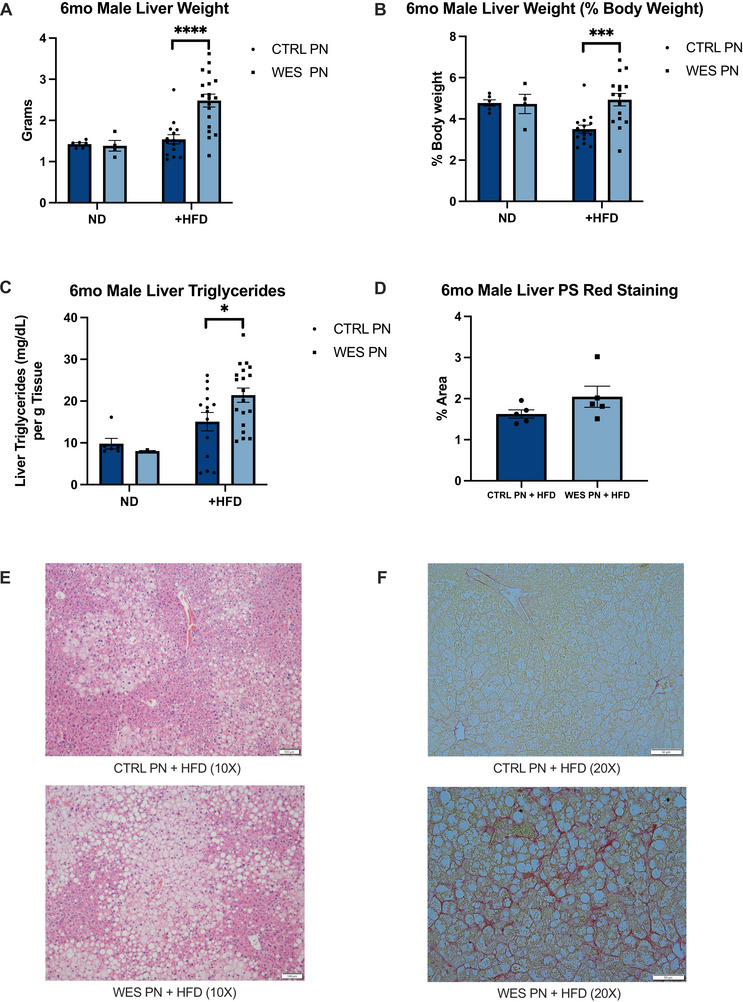
Male offspring exposed to a maternal Western‐style fat diet during lactation have increased liver weight and exhibit hepatic steatosis at 6 months of age. (A) Liver weights: CTRL PN + ND (*n* = 6), WES PN + ND (*n* = 4), CTRL PN + HFD (*n* = 15), WES PN + HFD (*n* = 19). (B) Liver weights as percentage of body weight. (C) Liver triglyceride concentration: CTRL PN + ND (*n* = 6), WES PN + ND (*n* = 3), CTRL PN + HFD (*n* = 14), WES PN + HFD (*n* = 19). (D) Liver fibrosis quantification by picro‐sirius red staining: CTRL PN + HFD (*n* = 5), WES PN + HFD (*n* = 5). (E) Representative histology by hematoxylin and eosin staining to visualize fat accumulation as open spaces. Bar represents 100 µm. (F) Representative images showing picro‐sirius red‐stained collagen fibers. Bar represents 50 µm. CTRL, control; WES, Western; ND, normal diet; HFD, high‐fat diet; PN, post‐natal. *p < 0.05, ***p < 0.001, ****p < 0.0001 (unpaired *t*‐test). Data are expressed as mean ± SEM.

Liver weights were measured at 6 months of age for all offspring. Within male offspring maintained on ND, there was no significant difference in liver weight between diet groups (*p* = 0.732 and *p* = 0.907, Figure 6A,B). There was also no significant difference in liver weight between CTRL PN +HFD and CTRL PN or WES PN offspring (*p* = 0.261, *p *= 0.250, Figure 6A,B). However, after HFD rechallenge, there was a significant 1.6‐fold increase in whole liver mass in WES PN +HFD compared to CTRL PN +HFD male offspring (*p* < 0.0001, Figure 6A). There was also a significant 1.4‐fold increase in liver mass as percentage of total body weight (*p* = 0.0004, Figure 6B). While there was no difference in hepatic triglyceride concentration between CTRL PN and WES PN offspring (*p* = 0.095), WES PN +HFD had a significantly higher triglyceride concentration when compared to CTRL PN +HFD offspring (*p* = 0.028, Figure 6C).

**FIGURE 7:**
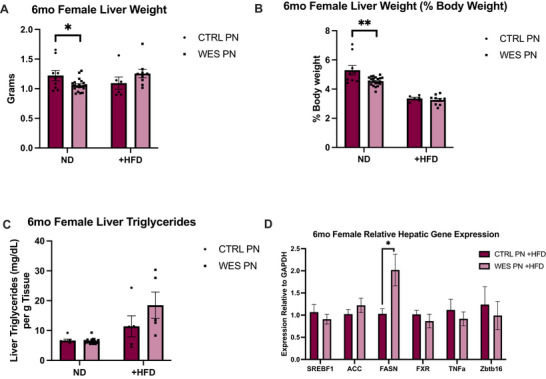
Female offspring liver weight, triglycerides, and gene expression at 6 months of age. (A) Liver weights: CTRL PN + ND (*n* = 9), WES PN + ND (*n* = 21), CTRL PN + HFD (*n* = 6), WES PN + HFD (*n* = 9). (B) Liver weights as percentage of body weight. (C) Liver triglyceride concentration: CTRL PN + ND (*n* = 7), WES PN + ND (*n* = 20), CTRL PN + HFD (*n* = 5), WES PN + HFD (*n* = 5). (D) Relative expression of genes in liver of 6‐month‐old female offspring. CTRL PN + HFD *n* = 5, WES PN + HFD *n* = 5 per gene. All replicates represent biological replicates. CTRL, control; WES, Western; ND, normal diet; HFD, high‐fat diet; PN, post‐natal. *p < 0.05, **p < 0.01, (unpaired *t*‐test). Data are expressed as mean ± SEM.

Within female offspring maintained on ND, WES PN had smaller liver weights in absolute weight and as percentage of total body weight (*p* = 0.018 and *p* = 0.0027, respectively, Figure 7A,B). After HFD rechallenge, there was no difference in liver weights between diet groups as absolute weight and percentage of total body weight (*p* = 0.205 and *p* = 0.527, respectively, Figure 7A,B). There was also no difference in hepatic triglyceride concentration between CTRL PN and WES PN offspring (*p* = 0.655, Figure  7C) and between CTRL PN +HFD and WES PN +HFD offspring (*p* = 0.245, Figure 7C), although values trended higher in the latter group.

**FIGURE 8:**
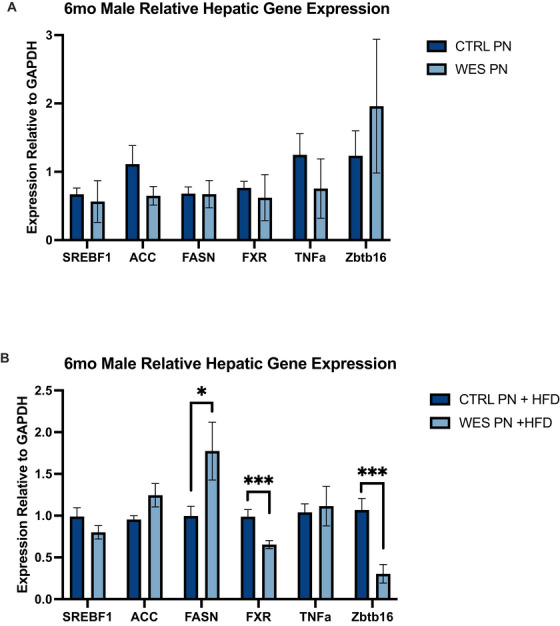
Hepatic gene expression at 6 months of age. (A). Relative expression of genes in liver of 6‐month‐old male offspring maintained on normal diet. CTRL PN *n* = 5 and 6, WES PN *n* = 5 per gene. (B) Relative expression of genes in liver of 6‐month‐old male offspring rechallenged with HFD. CTRL PN + HFD *n* = 10, WES PN +HFD *n* = 10 per gene. All replicates represent biological replicates. CTRL, control; WES, Western; HFD, high‐fat diet; PN, post‐natal. *p < 0.05, ***p < 0.001 (unpaired *t*‐test). Data are expressed as mean ± SEM.

We continued our liver morphology investigation in male offspring given the lack of differences in liver mass in female mice following HFD rechallenge. Male WES PN +HFD offspring developed evidence of hepatic steatosis. Pathologist review of hematoxylin and eosin‐stained liver specimens from CTRL PN +HFD offspring showed that 4/10 had evidence of hepatic steatosis, including 3/10 with grade 1 (mild) lesions and 1/10 with grade 2 (moderate). Additionally, 3/10 demonstrated evidence of steatohepatitis with grade 1 inflammation and ballooning. 0/10 demonstrated NAS scores of 5 or higher. Review of WES PN +HFD showed that 8/10 had evidence of hepatic steatosis, including 2/10 with grade 1 (mild) lesions and 6/10 with grade 2 (moderate). Furthermore, 4/10 demonstrated evidence of steatohepatitis with 3/4 demonstrating grade 1 inflammation and grade 2 ballooning and 1/4 demonstrating grade 2 inflammation. 3/10 demonstrated NAS scores of 5 or higher. There was no evidence of fibrosis in any specimen. Select images of these hematoxylin and eosin‐stained liver specimens are provided in Figure 6E. There was also a trend of increased percentage of collagen fibers stained with picro‐sirius red in male WES PN +HFD offspring, indicating increased liver fibrosis, although this was not statistically significant (*p* = 0.168, Figure 6D). Select images of these picro‐sirius red stained liver specimens are provided in Figure 6F.

### 3.5 Lactational Western‐Style Fat Exposure Leads to Changes in Hepatic Gene Expression After HFD Rechallenge in Male Offspring

To further understand the molecular events underlying the liver pathology observed in offspring, we analyzed the expression of genes involved in hepatic lipid metabolism, including fatty acid synthesis and oxidation, hepatic carbohydrate metabolism, and hepatic inflammation. Among male offspring maintained on ND, there was no difference in relative expression of the selected genes between CTRL PN and WES PN groups (Figure 8A). However, there was a significantly lower average relative expression of *Fxr* in WES PN +HFD offspring compared to CTRL PN +HFD (*p* = 0.0028, Figure 8B). Expression of *Zbtb16* was also significantly lower (*p* = 0.0008, Figure 8B). There was a significantly higher expression of *Fasn* in WES PN +HFD compared to CTRL PN +HFD (*p* = 0.0465, Figure 8B). The expression of *Acaca* trended upward among WES PN +HFD offspring (*p* = 0.0618, Figure 8B), while there was no significant difference in expression of *Srebf1* in WES PN +HFD compared to CTRL PN +HFD (*p* = 0.173, Figure 8B). There was also no significant difference in expression of *Tnf* (*p* = 0.773, Figure 8A) between +HFD groups. Among female offspring, there was a significantly higher average relative expression of *Fasn* in WES PN +HFD compared to CTRL PN +HFD (*p* = 0.03, Figure 8D). There was no significant difference in expression of *Srebf1* (*p* = 0.453), *Acaca* (*p* = 0.338), *Fxr* (*p* = 0.42), *Zbtb16* (*p* = 0.642), or *Tbf* (*p* = 0.507) between +HFD groups (Figure 8D).

## 4 Discussion

This study investigated the role of a maternal WES diet confined to the lactation period on the susceptibility of offspring to metabolic liver disease. During lactation, male WES PN offspring had heavier body, white adipose tissue, and liver weights, and higher serum insulin levels compared to CTRL PN. For mice that remained on standard chow through 6 months of age, no differences were observed in these parameters between diet groups, indicating that the offspring metabolic differences do not persist after weaning. However, when offspring were rechallenged with a “second‐hit” stressor of a HFD from 3 to 6 months of age, which is often used to reveal latently programmed phenotypes [12, 13, 20, 24], WES PN +HFD offspring had greater weight gain, fat mass, insulin resistance, liver weight, and hepatic triglyceride accumulation compared to CTRL PN +HFD. These findings were only observed in male offspring, suggesting that the developmental effects of a lactational WES are sexually dimorphic, with female offspring being relatively spared.

Female WES PN +HFD offspring had increased body weight at 6 months of age compared to CTRL PN +HFD offspring. Female WES PN +ND mice had increased fat mass and GWAT weight compared to CTRL PN +ND mice, but these changes were no longer significant in the HFD rechallenged mice. This suggests that female mice are more resilient to the early effects of lactational Western‐style fat exposure with an improved ability to cope with an obesogenic diet later in life. Accordingly, there was no evidence of hepatomegaly or hepatic triglyceride accumulation among female WES PN offspring in both the non‐rechallenged and HFD‐rechallenged groups. This is consistent with prior studies indicating that lactational HFD exposure predisposes male, but not female, offspring to later life obesity and MASLD [14, 25, 26]. Furthermore, in a study of Wistar rats, feeding mothers a cafeteria diet (low‐protein, high‐fat) led to greater fat mass and lower lean mass in offspring [27]. This effect was more persistent in male offspring, suggesting that female offspring may be less susceptible to fat accumulation induced by maternal obesogenic diets during lactation. The global prevalence of MASLD is higher among males than females, and clinically, male sex confers an increased MASLD risk [28, 29]. It is also possible that female mice may require a longer rechallenge period to unmask metabolic effects.

Adiposity is a significant risk factor for developing MASLD [30]. In this study, male WES PN +HFD offspring gained more weight by 6 months of age and had increased fat mass. In terms of specific fat depots, visceral fat is strongly linked to MASLD development [31]. GWAT, a visceral fat depot, was not increased in WES PN +HFD offspring; however, IWAT, a subcutaneous fat depot, was increased. MASLD has been independently associated with both visceral and subcutaneous adiposity in epidemiological studies [32, 33, 34] and prior mouse models utilizing a maternal HFD exposure during lactation similarly demonstrated increased IWAT but not GWAT weight among rechallenged offspring [12]. Mesenteric fat was not quantified in these studies and may be a more relevant visceral fat depot to compare with human epidemiologic risk. Collectively, exposure to maternal Western‐style fat during lactation programs offspring susceptibility to weight gain and adiposity linked to MASLD development when consuming an obesogenic diet in adulthood.

Insulin resistance is also associated with the development and progression of MASLD. In a state of insulin resistance, there is uninhibited lipolysis in adipose tissue and increased release of fatty acids into the circulation, which are taken up by the liver. Increased circulating insulin and glucose also stimulate hepatic de novo lipogenesis, contributing to hepatic steatosis [35]. Male WES PN +HFD offspring had increased serum insulin levels compared to CTRL PN +HFD. There was also a significantly diminished hypoglycemic response after insulin injection among these offspring, indicating insulin resistance. Glucose tolerance was unaffected. During the early stages of insulin resistance, tissues become less responsive to insulin but augmented pancreatic insulin secretion may allow for preservation of normal blood glucose levels. MASLD has been associated with hyperinsulinemia even in subjects with normal glucose tolerance [36, 37]. Furthermore, MASLD often precedes glucose dysregulation [38]. Taken together, exposure to maternal Western‐style fat during lactation increases offspring susceptibility to insulin resistance that may contribute to MASLD development.

Male WES PN +HFD offspring had enlarged livers compared to CTRL PN +HFD offspring. These offspring also had increased hepatic triglyceride accumulation, indicating hepatic steatosis. Histologically, livers demonstrated widespread steatosis and evidence of inflammation and ballooning without evidence of fibrosis. This suggests that compared to a HFD exposure, a maternal Western‐style fat exposure during lactation similarly programs metabolic liver disease susceptibility in offspring but without progression to more advanced forms of liver injury in this model [13, 14]. It would be important to understand if similar effects would be revealed with a Western‐style fat metabolic challenge, which would better model human consumption habits and is a limitation of this study. It is also possible that a MASH specific diet would reveal this susceptibility more than the HFD rechallenge employed here.

We found changes in hepatic gene expression that are consistent with the development of MASLD. Hepatic expression of the Farnesoid X receptor (*Fxr*) was reduced in male WES PN +HFD compared to CTRL PN +HFD offspring. FXR is a bile acid receptor that plays a crucial role in hepatic triglyceride homeostasis. Hepatic FXR expression is markedly reduced in obese animals and humans [39], and mice deficient in FXR demonstrate marked hepatic steatosis and hypertriglyceridemia [39]. FXR has been shown to play a role in regulating bile acid metabolism, hepatic lipogenesis, fatty acid uptake, fatty acid oxidation, and glucose homeostasis, which are all involved in the development of hepatic steatosis [40, 41]. Fatty acid synthase (*Fasn*) was found to be elevated in both male and female offspring after HFD challenge. *Fasn* catalyzes fatty acid synthesis, potentially correlating with the hepatic steatosis detected in the male offspring. While the females did not have a significant increase in liver triglycerides after HFD rechallenge, the mean triglyceride was 60% higher in the WES PN +HFD group. It is possible that this transcriptional signal suggests steatosis would be found with a longer exposure to HFD rechallenge in female WES PN +HFD offspring.

Promyelocytic leukemia zinc finger (PLZF), also known as *Zbtb16*, is a regulator of hepatic gluconeogenesis. We previously identified upregulation of *Zbtb16* expression in mice exposed to a maternal HFD during lactation, which has also been observed in multiple mouse models of diabetes [14]. Hepatic insulin resistance promotes gluconeogenesis, which promotes hepatic steatosis via lipogenesis [38]. While it seems counterintuitive that *Zbtb16* expression was downregulated in male WES PN+HFD offspring, it is possible that in the early stages of insulin resistance, intact insulin signaling suppresses gluconeogenesis while stimulating de novo lipogenesis [42]. Accordingly, fasting glucose levels were not elevated amongst offspring. On the other hand, it is possible that the downregulation is an attempt to compensate for the HFD stressor, as lower expression has been shown to improve hepatic steatosis in the setting of a dietary challenge [43].

In summary, we demonstrate that exposure to a maternal WES during lactation programmed male but not female offspring susceptibility to MASLD. We have identified potential molecular candidates that could be associated with the hepatic steatosis observed in this study. The unique aspect of this study is that a brief indirect exposure to a clinically relevant, maternal WES diet confined to the lactation period may contribute to lifelong metabolic disease and hepatic steatosis risk. This is potentially clinically relevant by providing support for lactation as a window of opportunity for targeting interventions, for example through dietary counseling for lactating parents. Future directions include better characterizing the mechanistic pathways underlying this phenomenon and exploring the roles of epigenetics and/or the gut microbiome in mediating gene expression changes. This could then allow for the development of targeted interventions for young offspring at risk for metabolic liver disease.

## Funding

This work was supported by R56 DK121787 (B.G.) and R01 DK107535 (D.B.). A.Z. was supported by the NIH Short‐Term Biomedical Research Training Program.

## Ethics Statement

The work herein was performed in compliance with the Institutional Animal Care and Use Committee (approval number: PRO00012189) and adheres to all ethical principles of research conduct.

## Conflicts of Interest

The authors declare no conflicts of interest.

## Data Availability

The data that support the findings of this study are available from the corresponding author upon reasonable request.
